# Clinical effects of natalizumab on multiple sclerosis appear early in treatment course

**DOI:** 10.1007/s00415-012-6809-7

**Published:** 2013-01-05

**Authors:** Ludwig Kappos, Paul W. O’Connor, Christopher H. Polman, Patrick Vermersch, Heinz Wiendl, Amy Pace, Annie Zhang, Christophe Hotermans

**Affiliations:** 1 Departments of Neurology and Biomedicine, University Hospital Basel, Petersgraben 4, Basel, Switzerland; 2 MS Clinic, St. Michael’s Hospital, 3 Suther Wing D. Room 3007, 30 Bond Street, Toronto, ON Canada; 3 VU Medical Centre, PO Box 7057, 1007 MB Amsterdam, The Netherlands; 4 Department of Neurology, Clinique neurologique, Hôpital Roger Salengro, University of Lille Nord de France, CHU de Lille, Rue Emile Laine, 59037 Lille Cedex, France; 5 Department of Neurology-Inflammatory Diseases of the Nervous System and Neurooncology, Neurology Clinic, University of Münster, Domagkstrasse 13, Munster, Germany; 6 Biogen Idec Inc., 133 Boston Post Road, Weston, MA 02493 USA

**Keywords:** Multiple sclerosis, Natalizumab, Relapse, Disease activity, Disease-modifying therapy, Annualized relapse rate

## Abstract

In clinical practice natalizumab is typically used in patients who have experienced breakthrough disease during treatment with interferon beta (IFNβ) or glatiramer acetate. In these patients it is important to reduce disease activity as quickly as possible. In a phase II study, differences between natalizumab and placebo in MRI outcomes reflecting inflammatory activity were evident after the first infusion and maintained through a 6-month period, suggesting a rapid onset of natalizumab treatment effects. To explore how soon after natalizumab initiation clinical effects become apparent, annualized relapse rates per 3-month period and time to first relapse were analyzed in the phase III AFFIRM study (natalizumab vs. placebo) and in the multinational Tysabri^®^ Observational Program (TOP). In AFFIRM, natalizumab reduced the annualized relapse rate within 3 months of treatment initiation compared with placebo in the overall population (0.30 vs. 0.71; *p* < 0.0001) and in patients with highly active disease (0.30 vs. 0.94; *p* = 0.0039). The low annualized relapse rate was maintained throughout the 2-year study period, and the risk of relapse in AFFIRM patients treated with natalizumab was reduced [hazard ratio against placebo 0.42 (95 % CI 0.34–0.52); *p* < 0.0001]. Rapid reductions in annualized relapse rate also occurred in TOP (baseline 1.99 vs. 0–3 months 0.26; *p* < 0.0001). Natalizumab resulted in rapid, sustained reductions in disease activity in both AFFIRM and in clinical practice. This decrease in disease activity occurred within the first 3 months of treatment even in patients with more active disease.

## Introduction

For patients with multiple sclerosis (MS), rapid control of disease activity is an important goal of therapy [1]. When clinical exacerbations are frequent and/or levels of radiologically apparent disease activity are high, effective treatment is especially crucial because the inflammation associated with active disease may lead not only to demyelination but also to potentially irreversible neuronal damage [2–6]. Disease-modifying therapies (DMTs) for the treatment of MS have been shown to reduce relapse rates and disability progression in pivotal studies over 1–2 years [7–11]. Clinical effects occurring earlier than 1 year after initiation of therapy have not yet been well studied. Some studies, however, have shown significant benefits on annualized relapse rates and/or the number of gadolinium-enhancing lesions with DMTs versus placebo at 6 months, suggesting that the clinical effects of therapy could be detectable at earlier time points as well [12–14].

Natalizumab (Tysabri^®^, Biogen Idec, Weston, MA, and Elan Pharmaceuticals, Inc., San Francisco, CA) is a recombinant humanized monoclonal antibody that inhibits binding of the α4 subunit of the α4β1 and α4β7 integrins to their endothelial receptors and prevents trafficking of mononuclear leukocytes across the vascular endothelium of the central nervous system (CNS) [15, 16]. In its pivotal monotherapy trial (AFFIRM), natalizumab showed efficacy at 1 and 2 years in treatment-naïve patients with relapsing forms of MS [15]. Analysis of 2-year data from AFFIRM revealed that natalizumab was also effective in the subgroup of patients with highly active disease, defined as having ≥2 relapses in the year before study entry and ≥1 gadolinium-enhancing lesion at study entry [17]. In additional studies, natalizumab was effective as a second-line therapy in patients with insufficient response to other DMTs [18–22].

Results from the phase II study suggest that natalizumab may reduce disease activity shortly after treatment initiation. At 1 month, the mean number of new gadolinium-enhancing lesions had already diverged between natalizumab- and placebo-treated patients, and the difference was maintained over time. At 6 months, there was an overall 89−93 % reduction of new gadolinium-enhancing lesions with natalizumab compared with placebo, as well as a significant reduction in the number of relapses [14].

To further explore when the effects of natalizumab on clinical relapses occur and whether the time course of clinical effects is dependent on the degree of baseline disease activity, we conducted post hoc analyses of data from AFFIRM. Because clinical practice likely has greater variability in patient characteristics compared with clinical trials, data from the clinical practice-based TYSABRI Observational Program (TOP) were also analyzed [23].

## Materials and methods

### Study design—AFFIRM

AFFIRM was a randomized, double-blind, placebo-controlled, phase III clinical study, in which 942 patients received natalizumab 300 mg or placebo (2:1) by intravenous (i.v.) infusion once every 4 weeks for up to 116 weeks [15]. Relapses were defined as new or recurrent neurologic symptoms not associated with fever or infection that lasted ≥24 h and were accompanied by new neurologic signs found by the examining neurologist. Post hoc subgroup analyses of data from AFFIRM defined highly active disease as ≥2 relapses in the year before study entry and ≥1 gadolinium-enhancing lesion on T1-weighted magnetic resonance imaging (MRI) at study entry [17].

### Study design—TOP

Tysabri^®^ Observational Program is an ongoing 10-year, open-label, multinational, multicenter, prospective observational study evaluating the long-term safety and efficacy of natalizumab in the postmarketing clinical practice setting. As of December 1, 2011, 3,976 patients were enrolled [24].

In TOP, a clinical relapse is defined as new or recurrent neurological symptoms, not associated with fever, lasting for at least 24 h, and followed by a period of 30 days of stability or improvement. New or recurrent neurological symptoms that occur less than 30 days following the onset of a protocol-defined relapse should be considered part of the same relapse.

AFFIRM and TOP were approved by the appropriate ethics committees and performed in accordance with the Declaration of Helsinki and Good Clinical Practice guidelines. All patients provided written informed consent.

### Statistical analysis

Analyses were conducted for overall AFFIRM and TOP populations and for the subgroup of AFFIRM patients with highly active disease. Annualized relapse rates per 3-month interval were calculated as the total number of relapses divided by total person-years observed within the interval. Point estimates, confidence intervals (CIs), and treatment effects (rate ratios) for each interval were estimated from a Poisson model with overdispersion. For TOP data, annualized relapse rates were estimated at each time point using a negative binomial model. Treatment effects on time to first relapse (in days) at a specific time point were assessed using hazard ratios (HR) estimated from a Cox proportional hazards model, adjusting for the number of relapses in the year before study entry. The cumulative probability of relapse was estimated using the Kaplan–Meier method, and 95 % confidence bands were derived using the equal precision approach [25]. A log-rank test was used to determine the first day a significant difference in the cumulative probability of relapse emerged between study groups.

## Results

### Baseline relapse rates

Relapse rates at baseline for patients in AFFIRM and TOP are shown in Table 1. Overall, the mean baseline relapse rate in AFFIRM was 1.5 relapses in the prior year. A total of 209 patients (148 natalizumab and 61 placebo) in AFFIRM met the criteria for highly active MS, with a mean relapse rate of 2.5 relapses in the year prior to study entry.

**Table 1:** Baseline annualized relapse rates for patients in AFFIRM and TOP

	AFFIRM				TOP
	Natalizumab		Placebo		Natalizumab
	Overall	HA	Overall	HA	Overall
Patients, *n*	627	148	315	61	3,963^a^
Relapses during prior year, *n*					
Mean ± SD	1.53 ± 0.91	2.45 ± 1.19	1.50 ± 0.77	2.28 ± 0.55	1.99 ± 1.03
95 % CI	1.46–1.60	2.25–2.64	1.42–1.59	2.14–2.42	1.96–2.03
Median (range)	1 (0–12)	2 (2–12)	1 (0–5)	2 (2–4)	2 (0–10)

*HA* highly active, defined as having ≥2 relapses in the year before study entry and ≥1 gadolinium-enhancing lesion at study entry

^a^Baseline relapse rates were not available for 13 patients in TOP

The median (range) disease duration prior to starting natalizumab was similar for the two studies: 5 (0–34) years for AFFIRM and 7 (0–44) years for TOP [15, 23]. The majority (90 %) of TOP patients had used another DMT prior to starting natalizumab [23]. The mean relapse rate at baseline among TOP patients was approximately 2.0, similar to the rate in AFFIRM patients with highly active disease (Table 1).

### Annualized relapse rate

**Fig. 1:**
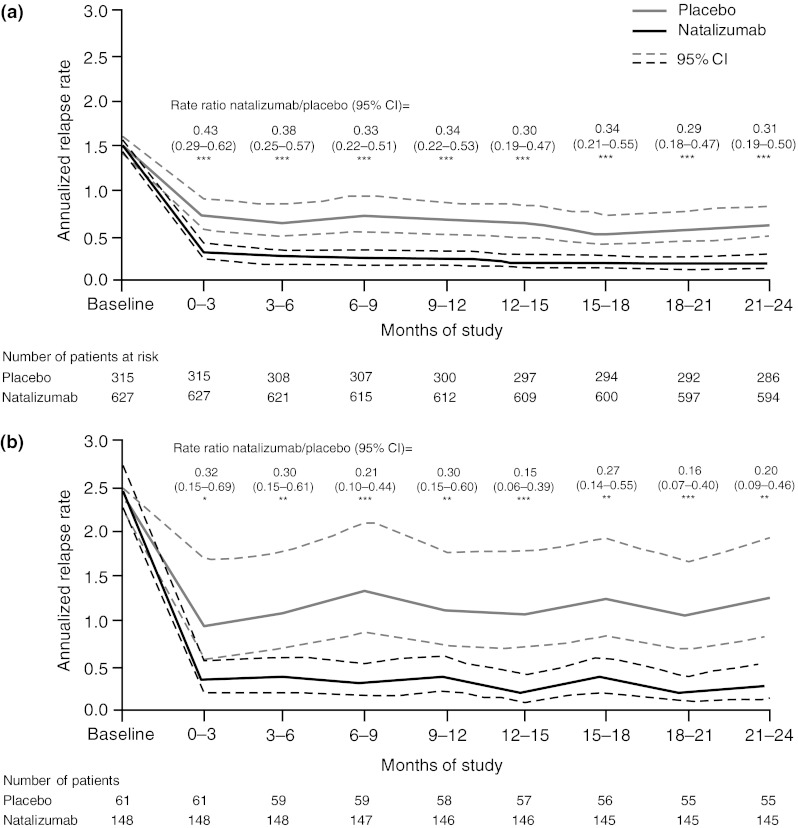
Annualized relapse rate over time (calculated for each 3-month interval) in AFFIRM patients overall (**a**) and with highly active disease (**b**) is shown for placebo-treated (*solid gray line*) and natalizumab-treated (*solid black line*) patients; 95 % CI are indicated by *dashed lines*. Rate ratios of natalizumab/placebo (95 % CI) for each time period are indicated in the graphs. *p < 0.01; **p < 0.001; ***p < 0.0001

In AFFIRM natalizumab monotherapy reduced annualized relapse rate already within the first 3 months of treatment compared with placebo overall [0.30 (95 % CI 0.23–0.40) vs. 0.71 (95 % CI 0.55–0.91); *p* < 0.0001] and in patients with highly active disease [0.30 (95 % CI 0.17–0.53) vs. 0.94 (95 % CI 0.55–1.63); *p* = 0.0039]. This treatment effect was maintained throughout the 2-year controlled study period and was observed despite the fact that sizeable reductions from baseline annualized relapse rate occurred also in the placebo group (Fig. 1).

**Fig. 2:**
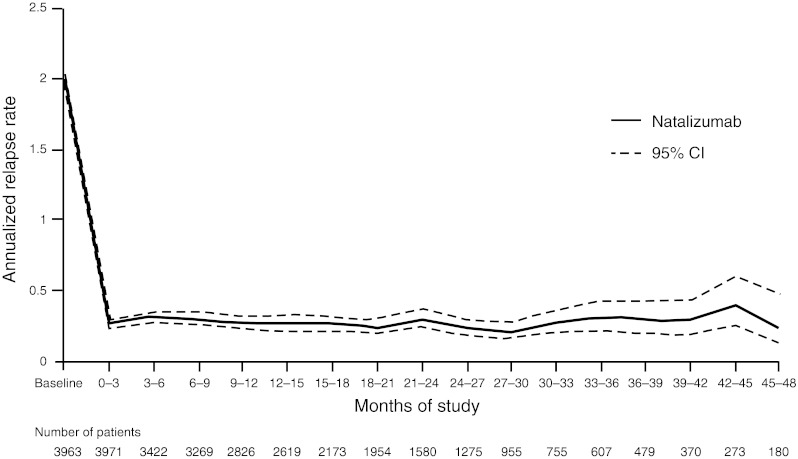
Annualized relapse rate over time in TOP patients is shown as a solid line; 95 % CIs are indicated by *dashed lines*. Five patients did not receive natalizumab and were excluded from post-baseline analyses

In TOP, annualized relapse rate was reduced from 1.99 (95 % CI 1.96–2.03) at baseline to 0.26 (95 % CI 0.23–0.30; *p* < 0.0001) after 3 months of natalizumab treatment and was maintained at that rate at 4 years (Fig. 2).

### Time to first relapse

**Fig. 3:**
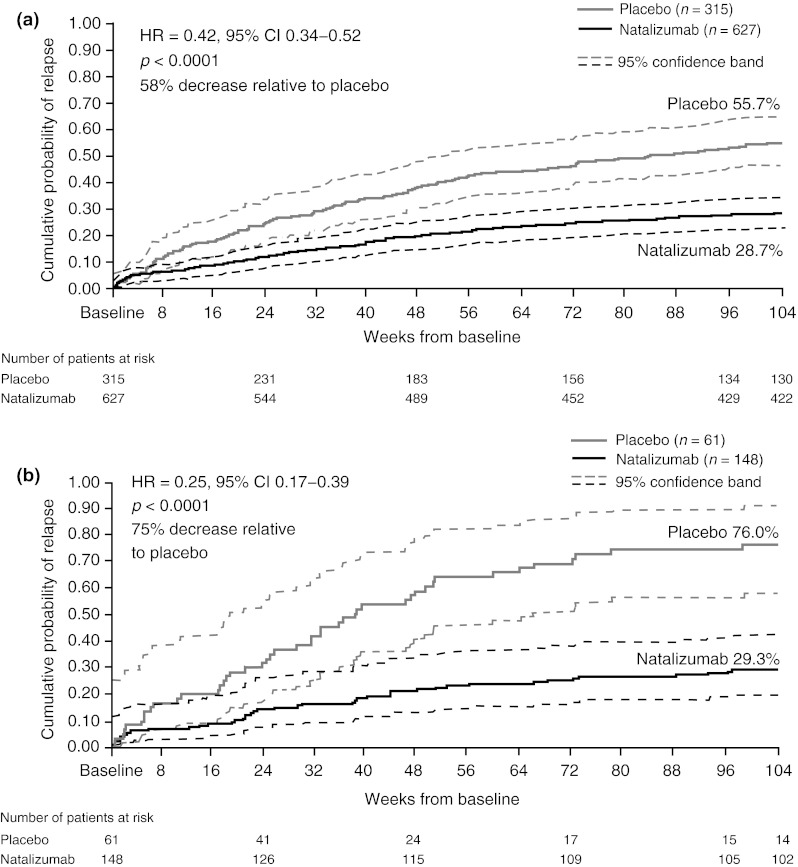
Cumulative probability of relapse in AFFIRM patients overall (**a**) and with highly active disease (**b**) is shown for placebo-treated (*gray line*) and natalizumab-treated (*black line*) patients

Kaplan–Meier curves estimating the cumulative probability of relapse over 2 years in AFFIRM patients overall and in those with highly active disease are shown in Fig. 3. A significant treatment effect comparing natalizumab versus placebo was observed at 8 weeks after initiating treatment (Table 2). A difference in the cumulative probability of relapse from baseline between the two groups was first observed at day 42 in patients overall, 5.4 % for natalizumab and 9.3 % for placebo (HR: 0.56, 95 % CI 0.34–0.93; *p* = 0.0238), and at day 45 in patients with highly active disease, 6.8 % for natalizumab and 16.6 % for placebo (HR: 0.35, 95 % CI 0.14–0.87; *p* = 0.0243). The treatment effect was maintained at 2 years. Overall, natalizumab treatment reduced the risk of relapse by 58 % over 2 years relative to placebo (*p* < 0.0001). In patients with highly active disease, natalizumab reduced the risk of relapse by 75 % over 2 years relative to placebo (*p* < 0.0001).

**Table 2:** Cumulative risk of relapse from baseline for patients in AFFIRM

	Overall		HA	
	Natalizumab	Placebo	Natalizumab	Placebo
Cumulative risk of relapse at 4 weeks from baseline				
Percentage	4.6	4.8	6.1	8.3
HR (95 % CI)	0.96 (0.51–1.79)		0.72 (0.24–2.16)	
* p* value	0.8893		0.5578	
Cumulative risk of relapse at 8 weeks from baseline				
Percentage	5.7	12.2	6.8	16.6
HR (95 % CI)	0.45 (0.29–0.72)		0.35 (0.14–0.87)	
* p* value	0.0007		0.0243	
Cumulative risk of relapse at 12 weeks from baseline				
Percentage	7.2	16.7	7.4	20.0
HR (95 % CI)	0.41 (0.28–0.61)		0.33 (0.14–0.76)	
* p* value	<0.0001		0.0090	
Cumulative risk of relapse at 24 weeks from baseline				
Percentage	11.9	25.4	14.2	33.3
HR (95 % CI)	0.43 (0.31–0.59)		0.37 (0.20–0.68)	
* p* value	<0.0001		0.0015	
Cumulative risk of relapse at 48 weeks from baseline				
Percentage	20.0	39.2	21.7	58.9
HR (95 % CI)	0.45 (0.35–0.57)		0.28 (0.17–0.45)	
* p* value	<0.0001		<0.0001	
Cumulative risk of relapse at 104 weeks from baseline				
Percentage	28.7	55.7	29.3	76.0
HR (95 % CI)	0.42 (0.34–0.52)		0.25 (0.17–0.39)	
* p* value	<0.0001		<0.0001	

*HA* highly active, defined as having ≥2 relapses in the year before study entry and ≥1 gadolinium-enhancing lesion at study entry

**Fig. 4:**
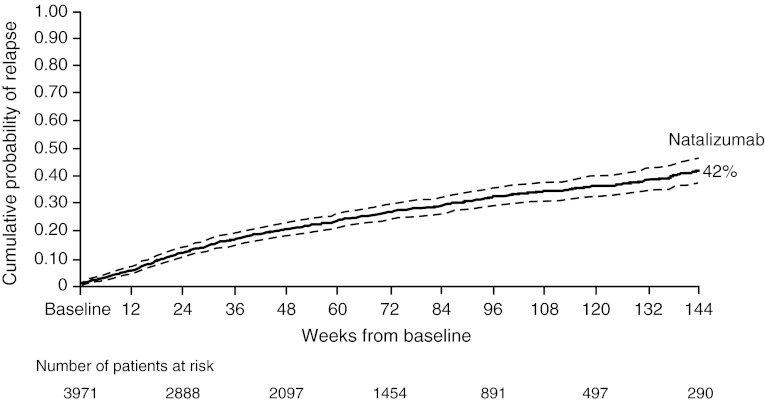
Cumulative probability of relapse in TOP patients is shown as a *solid line*; 95 % confidence band is indicated by *dashed lines*. Five patients did not receive natalizumab and were excluded from post-baseline analyses

At 4, 8, and 12 weeks in TOP, patients treated with natalizumab had an estimated 2.4, 4.0, and 5.7 % cumulative risk of relapse from baseline, respectively. At 2 years, the estimated risk was 32 %, similar to the risk in natalizumab-treated AFFIRM patients at 2 years. At 3 years in TOP, the cumulative risk of relapse was 42 % (Fig. 4).

## Discussion

In AFFIRM, natalizumab had a significant effect on annualized relapse rate within 3 months of initiating therapy, and reductions were sustained over the 2-year controlled study period. Combined with the MRI findings of the phase II study in which reductions in the mean number of new gadolinium-enhancing lesions in natalizumab-treated patients versus placebo were apparent after 1 month and remained reduced as long as treatment was continued [14], these data suggest that natalizumab reduces disease activity shortly after initiating treatment.

Natalizumab also had a significant effect on the cumulative probability of relapse over 2 years, with significant reductions being observed by 8 weeks after starting treatment. These rapid, sustained reductions in annualized relapse rate and risk of relapse with natalizumab treatment occurred regardless of baseline disease activity in the overall population and in the subset of patients with highly active disease.

Observations from a clinical practice setting (TOP) were similar to the clinical trial results but should be interpreted with caution owing to the lack of a reference group. Nevertheless, given the differences in patient demographics and disease characteristics between the clinical studies and TOP, these results suggest that the results from the pivotal studies are applicable to those patients included in the current label of natalizumab. This is particularly remarkable regarding the potential utility of natalizumab for patients experiencing MS disease activity despite treatment with other DMTs to rapidly gain control of disease and help prevent further cumulative damage.

Our findings are consistent with those of numerous post-marketing studies that have observed significant improvements in annualized relapse rate, radiologic disease, ambulation, or disability progression in patients, including those with highly active disease, switching to natalizumab from other DMTs, although most assessments were performed at 1 year after initiating natalizumab [18, 19, 21, 22, 26–32]. The results of this analysis are also consistent with a separate AFFIRM analysis that showed an increased probability of sustained improvement in Extended Disability Status Scale (EDSS) scores with natalizumab compared with placebo emerging during the first 24 weeks of treatment [33].

Natalizumab’s rapid effects and ability to control disease in patients with highly active MS may be best explained by the inhibition of leukocyte migration into brain tissue that prevents lesion formation and reduces inflammatory cell recruitment into existing lesions [34, 35]. In addition to preventing leukocyte entry into the CNS, there is evidence that natalizumab may dampen ongoing inflammation in the CNS by disrupting interactions between inflammatory leukocytes and extracellular matrix proteins such as fibronectin and osteopontin or by inducing apoptosis of activated T cells [36–38].

In summary, rapid onset of natalizumab clinical efficacy shortly after treatment initiation may provide both short- and long-term benefits.
